# Supplementary material for: A combination treatment based on drug repurposing demonstrates mutation-agnostic efficacy in pre-clinical retinopathy models
Source: Nat Commun. 2024 Jul 15;15:5943. doi: 10.1038/s41467-024-50033-5 (PMC11251169; doi:10.1038/s41467-024-50033-5)
Supplement: Supplementary file 1 — Supplementary information [file 41467_2024_50033_MOESM1_ESM.docx]

SUPPLEMENTARY INFORMATION

|  | **A combination treatment based on drug repurposing demonstrates mutation-agnostic efficacy in pre-clinical retinopathy models** |
| --- | --- |

Henri Leinonen^1*^, Jianye Zhang^2^, Laurence M. Occelli^3^, Umair Seemab^1^, Elliot H. Choi^2^, Luis Felipe L.P. Marinho^3^, Janice Querubin^3^, Alexander V. Kolesnikov^2^, Anna Galinska^4,5^, Katarzyna Kordecka^4,5^, Thanh Hoang^6^, Dominik Lewandowski^2^, Timothy T. Lee^2^, Elliott E. Einstein^2^, David E. Einstein^2^, Zhiqian Dong^2^, Philip D. Kiser^2,7,8,9^, Seth Blackshaw^10,11,12,13^, Vladimir J. Kefalov^2,7^, Marcin Tabaka^4,5^, Andrzej Foik^4.5^, Simon M. Petersen-Jones^3^, Krzysztof Palczewski^2,7,14,15*^

^1^School of Pharmacy, Faculty of Health Sciences, University of Eastern Finland, Yliopistonranta 1C, 70211 Kuopio, Finland. Email: henri.leinonen@uef.fi

^2^Gavin Herbert Eye Institute-Center for Translational Vision Research, Department of Ophthalmology, University of California, Irvine, CA, 92697, USA.

^3^Small Animal Clinical Sciences, Michigan State University, East Lansing, MI 48824, USA.

^4^International Centre for Translational Eye Research, Warsaw, Poland.

^5^Institute of Physical Chemistry, Polish Academy of Sciences, Warsaw, Poland.

^6^Department of Ophthalmology, Department of Cell & Developmental Biology, Ann Arbor, MI 48105, USA.

^7^Department of Physiology and Biophysics, School of Medicine, University of California - Irvine, Irvine, California 92697, USA.

^8^Department of Clinical Pharmacy Practice, School of Pharmacy and Pharmaceutical Sciences, University of California - Irvine, Irvine, California 92697, USA.

^9^Research Service, VA Long Beach Healthcare System, Long Beach, California 90822, United States.

^10^Department of Ophthalmology, Johns Hopkins University School of Medicine, Baltimore, MD 21205, USA.

^11^Department of Neurology, Johns Hopkins University School of Medicine, Baltimore, MD 21205, USA.

^12^Institute for Cell Engineering, Johns Hopkins University School of Medicine, Baltimore, MD 21205, USA.

^13^Kavli Neuroscience Discovery Institute, Johns Hopkins University School of Medicine, Baltimore, MD 21205, USA.

^14^Department of Chemistry, University of California-Irvine, Irvine, CA 92697, USA.

^15^Department of Molecular Biology and Biochemistry, University of California-Irvine, Irvine, CA 92697, USA.

*To whom **correspondence** should be addressed: Henri Leinonen, email: [henri.leinonen@uef.fi](mailto:henri.leinonen@uef.fi), phone: +358504303196; Krzysztof Palczewski, email: [kpalczew@uci.edu](mailto:kpalczew@uci.edu), phone: +19498246527

Table of Contents

[Supplementary Figure 1. Control experiments regarding dietary TMB administration. 3](#_Toc168609369)

[Supplementary Figure 2. Full western blots from vivarium-reared rd10 mouse retinas. 4](#_Toc168609370)

[Supplementary Figure 3. TMB treatment retains efficacy in rd10 mice with 5-fold lower doses compared to the standard doses used throughout the study. 5](#_Toc168609371)

[Supplementary Figure 4. Comparison of low TMB and high TMB treatment effect on M cone function in rd10 mice. 6](#_Toc168609372)

[Supplementary Figure 5. Full western blots from dark-reared rd10 mouse retinas. 7](#_Toc168609373)

[Supplementary Figure 6. Enlarged images of flat mounts. 8](#_Toc168609374)

[Supplementary Figure 7. Single-cell transcriptome UMAPs separately for each group in the rd10 mouse dataset. 9](#_Toc168609375)

[Supplementary Figure 8. Representative OCT and retinal flat-mount images from the study of the Rpe65-/- mouse. 10](#_Toc168609376)

[Supplementary Figure 9. Bulk RNA-seq in Rpe65-/- and WT retinas indicates transcriptomic stabilization closer to healthy levels with TMB treatment. 11](#_Toc168609377)

[Supplementary Figure 10. Single-cell RNA sequencing in Rpe65-/- mouse retinas indicate extensive transcriptomic regulation particularly in the Müller glia cells. 12](#_Toc168609378)

[Supplementary Figure 11. Single-cell transcriptome UMAPs separately for each group in the Rpe65-/- mouse dataset. 13](#_Toc168609379)

[Supplementary Figure 12. TMB treatment enhances mitochondrial gene expression in Rpe65-/- mouse rods and RBCs, and improves rod-mediated ERG responses. 14](#_Toc168609380)

[Supplementary Figure 13. Drug efficacy-evaluation parameters do not differ substantially between dog litters. 15](#_Toc168609381)

[Supplementary Figure 14. ERG waveforms in heterozygote PDE6A carrier dogs (A), and in homozygote PDE6A dogs during the first and last recording session. 16](#_Toc168609382)

[Supplementary Figure 15. PNA puncta-counting method for flat mounts of dog retinas. 17](#_Toc168609383)

[Supplementary Figure 16. Dorsal and ventral middle-retina images, for dog flat mounts. 18](#_Toc168609384)

[Supplementary Figure 17. Area centralis images from dog flat mounts. 19](#_Toc168609385)

[Supplementary Table 1. List of antibodies used in the study. 20](#_Toc168609386)

[Supplementary Table 2. The detection of drugs with mass spectrometry. 21](#_Toc168609387)


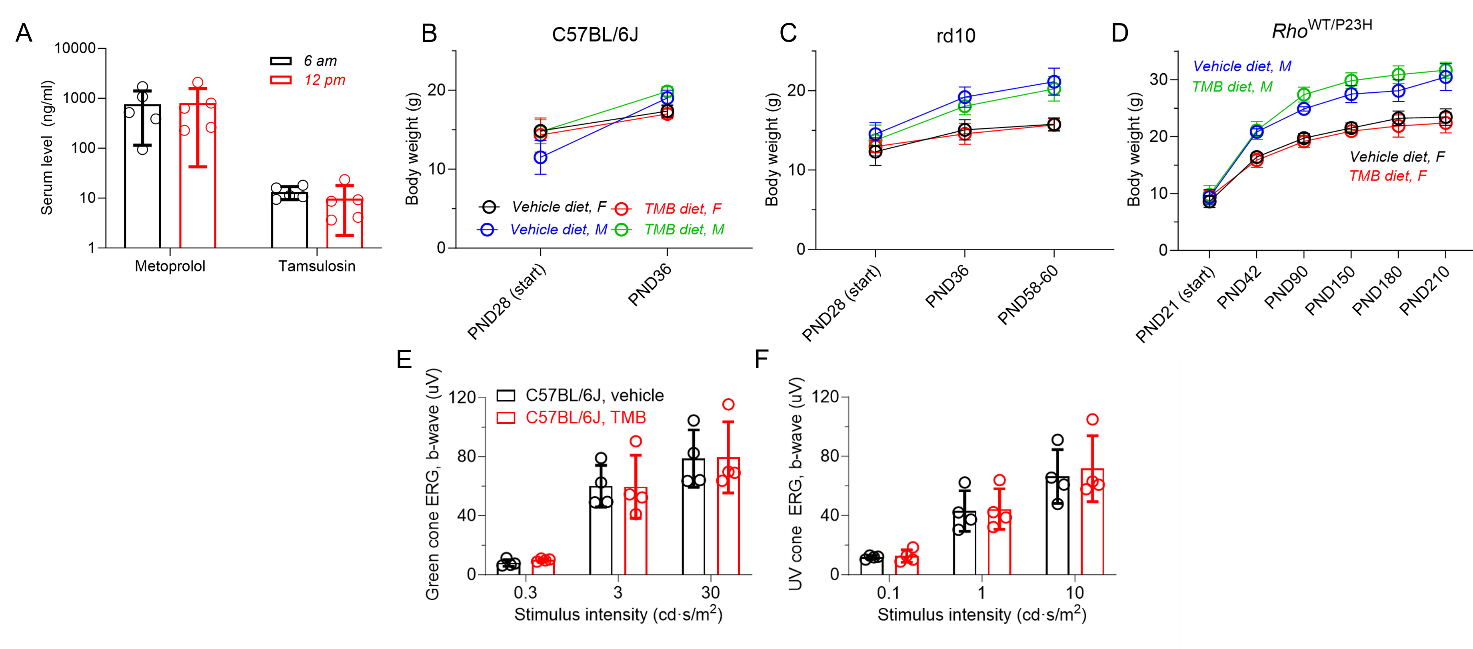


Supplementary Figure 1. Control experiments regarding dietary TMB administration. Data relates to main Figures 1-8. (**A**) Drug serum levels in samples collected at 6 a.m. or 12 p.m. Doses in the drug pellets were: tamsulosin 50 parts per million (ppm) / 50 mg compound per 1 kg of pellet, metoprolol 2500 ppm / 2500 mg compound per 1 kg of pellet, bromocriptine 250 pm / 250 mg compound per 1 kg of pellet. (**B**-**D**) Dietary TMB did not affect body weight gain in (**B**) wild-type (WT) C57BL/6J, (**C**) rd10, (**D**) or *Rho*^WT/P23H^ mice. Body weight gain is one of the best measures of general health in laboratory rodents. (**E**-**F**) One-month-long dietary TMB administration did not alter ERG responses in WT mice. Data in B-F were analyzed by two-way RM ANOVA with Geisser-Greenhouse correction. All data are presented as mean ± SD.

**
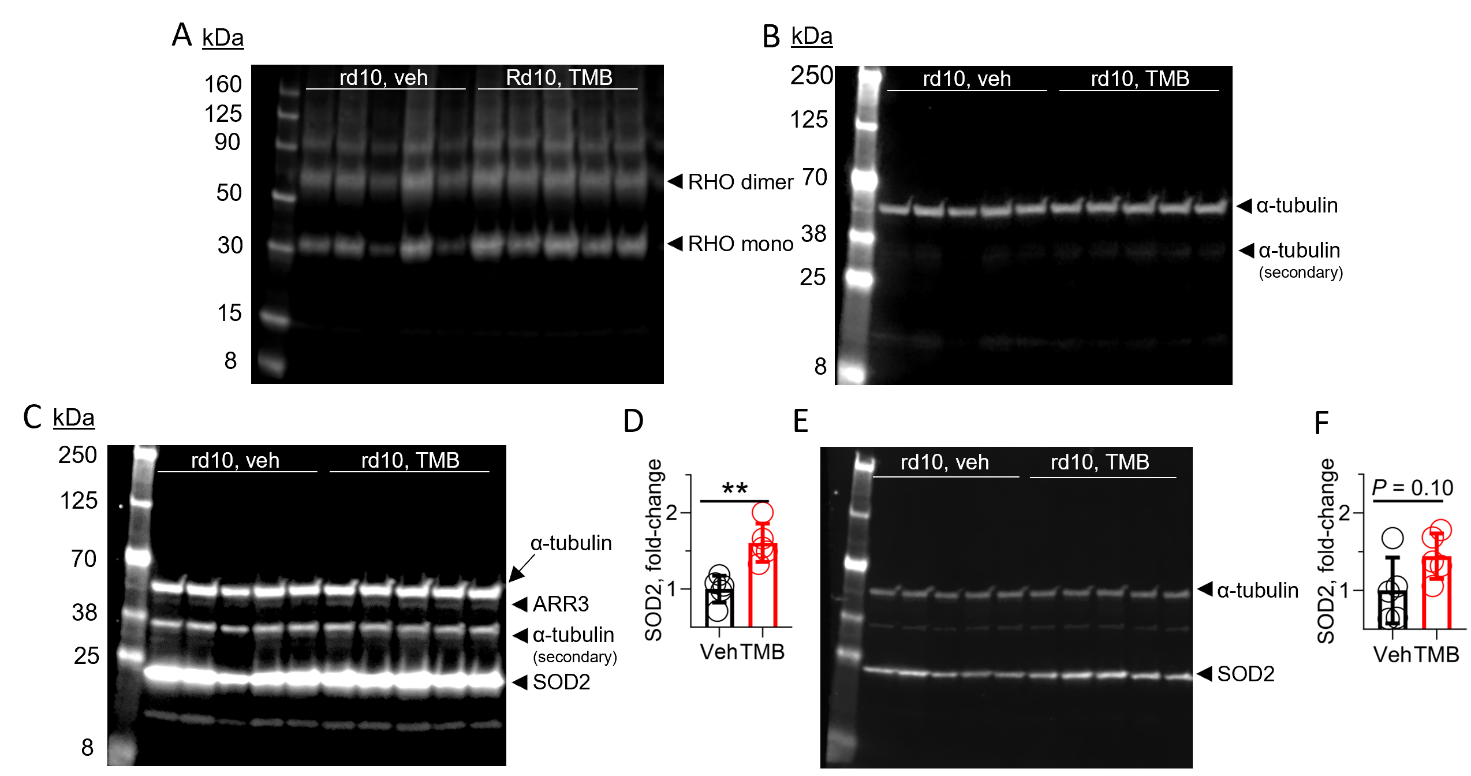
**

Supplementary Figure 2. Full western blots from vivarium-reared rd10 mouse retinas. Cone-arrestin and rhodopsin data was quantified and used in main Figure 2L. 40 µg of protein was loaded to detect rhodopsin (RHO), cone-arrestin (ARR3) and α-tubulin (TUBA1A). (**A**) Mouse monoclonal 1D4 antibody (1:2000 dilution) yields several RHO bands in mouse retinas. RHO signal was detected using LI-COR IRdye 680RD secondary antibody. (**B**) α-tubulin signal (1:2000 dilution) was detected using LI-COR IRdye 800CW. (**C**) The same membrane was re-incubated overnight in antibodies against ARR3 (1:2000 dilution) and superoxide dismutase 2 (SOD2, 1:5000 dilution). (**D**) Normalized SOD2 expression as detected from 40 ug protein-loaded membranes. Welch´s t-test: ***P* < 0.01. (**E**) SOD2 signal was also inspected with a lower protein load of 8 µg. (**F**) Normalized SOD2 expression as from 8 ug protein-loaded membranes. Immunoblotting experiments were not repeated. Data are presented as mean ± SD.


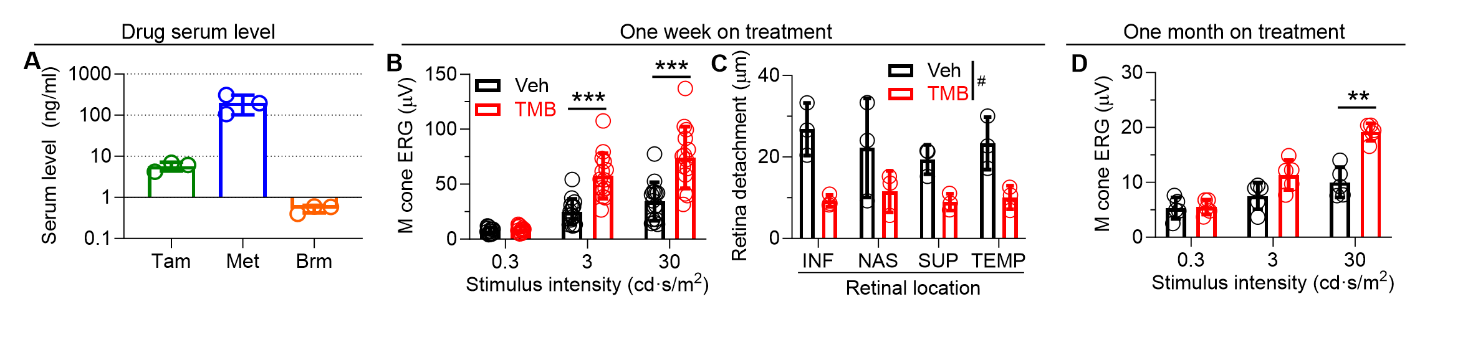


Supplementary Figure 3. TMB treatment retains efficacy in rd10 mice with 5-fold lower doses compared to the standard doses used throughout the study. Doses of drug in the food pellets in this case were: tamsulosin, 10 ppm (10 mg compound per 1 kg of pellets); metoprolol, 500 ppm (500 mg compound per 1 kg of pellets); bromocriptine, 50 ppm (50 mg compound per 1 kg of pellets). The study design was the same as in main Figure 1. (**A**) Drug levels in serum as assayed from samples collected at 6 a.m. (**B**) M-cone-dominant photopic ERG amplitudes after one week of treatment. (**C**) Retinal detachment in the central retina (500 μm from ONH) after one week of treatment, as measured from OCT images. (**D**) M-cone-dominant photopic ERG amplitudes after one month of treatment. Data in B-D were analyzed using two-way RM ANOVA with Geisser-Greenhouse correction and followed by Bonferroni post hoc tests. The pound sign signifies ANOVA between-subjects main result: ^#^ *P* < 0.05. The asterisks signify results from the post hoc tests: ***P* < 0.01, ****P* < 0.001. Data are presented as mean ± SD.

***
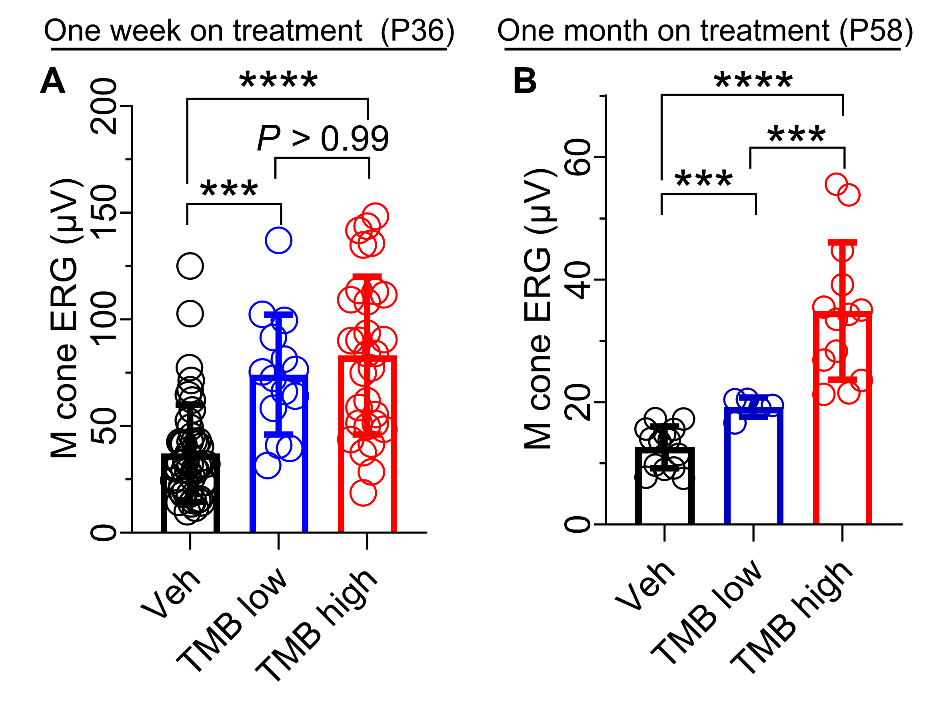
***

Supplementary Figure 4. Comparison of low TMB and high TMB treatment effect on M cone function in rd10 mice. The study design was the same as in main Figure 1. Data is reproduced from Figures 1G, 1W, S3B, and S3D. Response amplitudes to green flash stimulus at 30 cd·s/m^2^ are shown. Vehicle-group data is pooled from all experiments. (**A**) Response amplitudes after one week, or (**B**) after one month on TMB treatments. Doses in the TMB low pellets were: tamsulosin 10 parts per million (ppm) / 10 mg compound per 1 kg of pellet, metoprolol 500 ppm / 500 mg compound per 1 kg of pellet, bromocriptine 50 pm / 50 mg compound per 1 kg of pellet. Doses in the TMB high pellets were: tamsulosin 50 ppm, metoprolol 2500 ppm, bromocriptine 250 pm. Data in A were analyzed by Kruskal-Wallis test followed by Dunn´s post hoc tests. Data in B were analyzed by Welch´s ANOVA followed by Dunnett´s T3. The asterisks signify results from the post hoc tests: ****P* < 0.001, *****P* < 0.0001. Data are presented as mean ± SD.

**
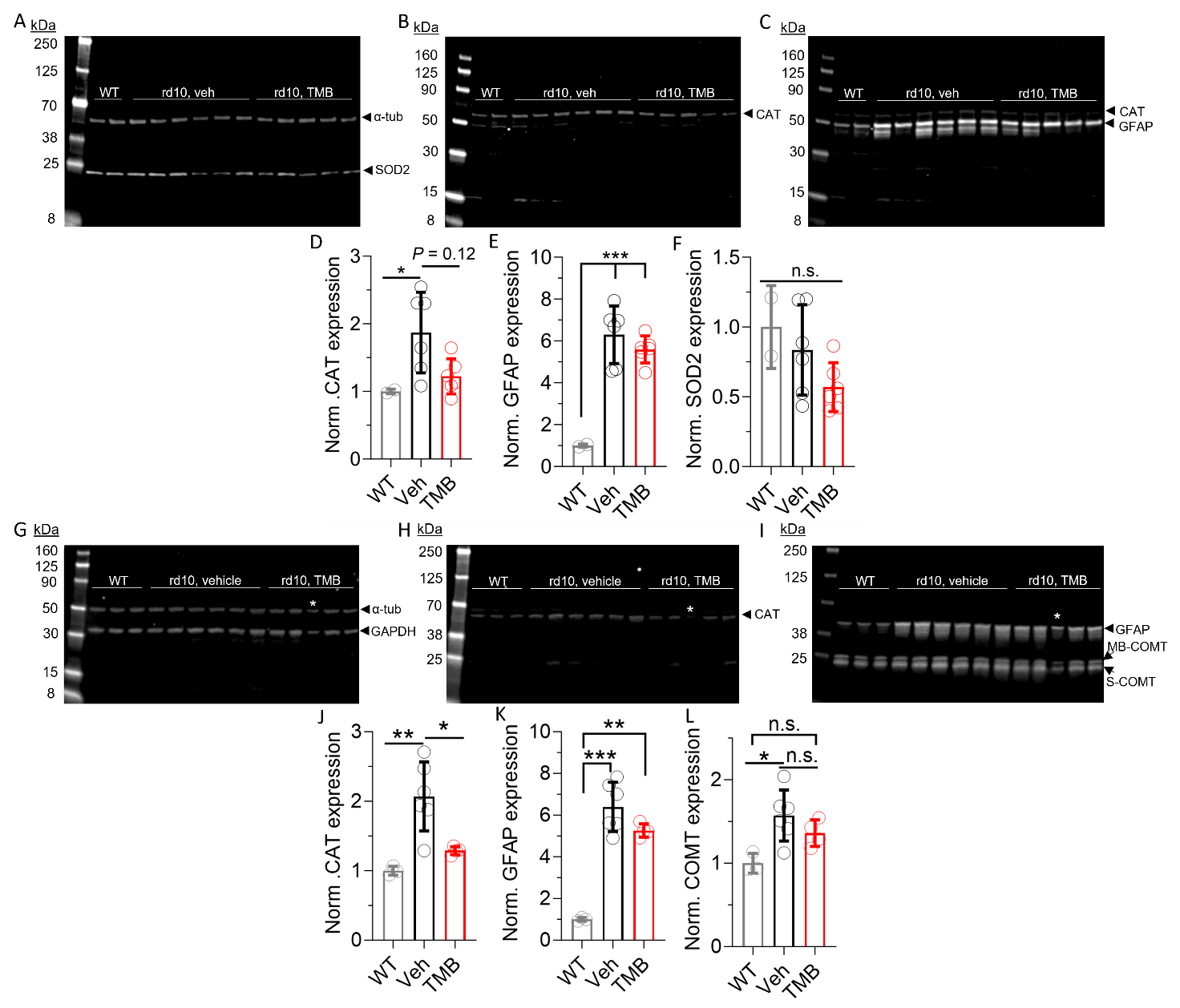
**

Supplementary Figure 5. Full western blots from dark-reared rd10 mouse retinas. Data relates to Figure 3I-K. Data in panels A-F and G-L display results from two individual cohorts/experiments. 40 µg of protein was loaded to detect α-tubulin (TUBA1A, 1:2000 dilution), superoxide dismutase 2 (SOD2, 1:5000 dilution), catalase (CAT, 1:500 dilution), glial acidic fibrillary protein (GFAP, 1:5000 dilution), and catechol-O-methyltransferase (COMT, 1:2000 dilution). (**A**) α-tubulin and SOD2 bands were detected using LI-COR IRdye 800CW. (**B**) The CAT band was detected using LI-COR IRdye 680RD secondary antibody. (**C**) The same membrane was re-incubated overnight in a solution containing antibody against GFAP, and the signal was detected using LI-COR IRdye 680RD antibody. (**D**-**F**) Relative expression levels of CAT, GFAP, and SOD2 were normalized to the level of α-tubulin, and contrasted with the WT level. (**G**) α-tubulin and glyceraldehyde-3-phosphate dehydrogenase (GAPDH) bands were detected using LI-COR IRdye 800CW. GAPDH was used in this experiment as a secondary loading control. Lane marked with the asterisk * was omitted from quantitative analysis due to low protein load, or sample degradation. (**H**) The CAT band was detected using LI-COR IRdye 680RD antibody. (**I**) The same membrane was re-incubated overnight in a solution containing antibodies against GFAP and COMT, and the respective bands were detected using LI-COR IRdye 680RD antibody. (**J**-**L**) Relative expression levels of CAT, GFAP, and COMT were normalized to the level of α-tubulin, and contrasted with the WT level. Quantification of membrane-bound (MB) and soluble (S) COMT was combined for analysis in panel L. Statistical analyses in graphs D and K were performed by Welch´s ANOVA followed by Dunnett´s T3 tests, whereas regular ANOVA and Bonferroni post hoc tests was used in graphs E, F, J and L. The asterisks signify results from post hoc tests: **P* < 0.05, ***P* < 0.01, ***P* < 0.001. Immunoblotting experiments were not repeated. Data are presented as mean ± SD. Quantified CAT and GFAP expression data was from the two cohorts was used as consolidated data in main Figure 3I-J.

**
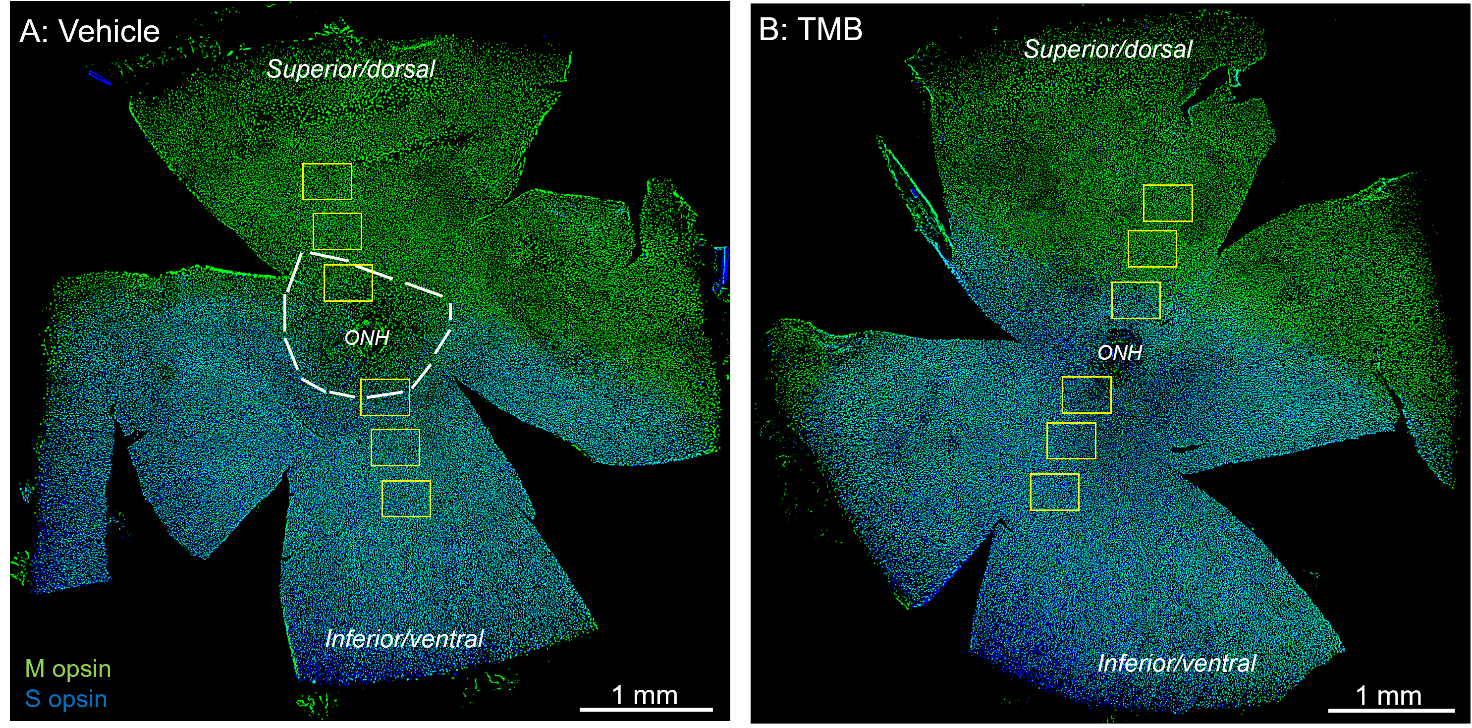
**

Supplementary Figure 6. Enlarged images of flat mounts, from main Figure 3 L-M. Dashed white line circling the optic nerve head (ONH) in panel A highlights evident degeneration in cone-population density. Yellow squares depict counting windows used for evaluation of the cone populations in main Figure 3, panels N and O.

**
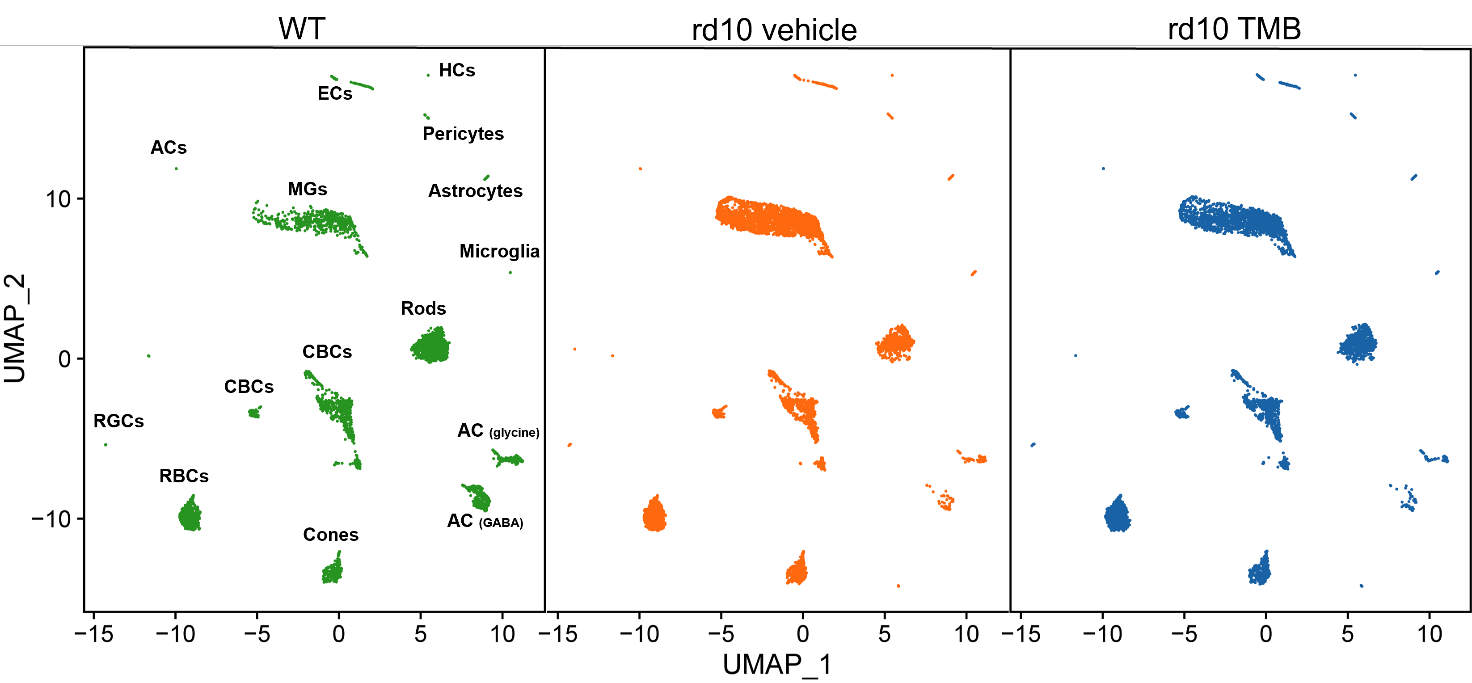
**

Supplementary Figure 7. Single-cell transcriptome UMAPs separately for each group in the rd10 mouse dataset. Figure relates to main Figure 4. RBCs, rod bipolar cells; CBC, cone bipolar cells; ACs, amacrine cells; ECs, endothelial cells; HCs, horizontal cells; MGs, Müller glia cells; RGCs, retinal ganglion cells.

**
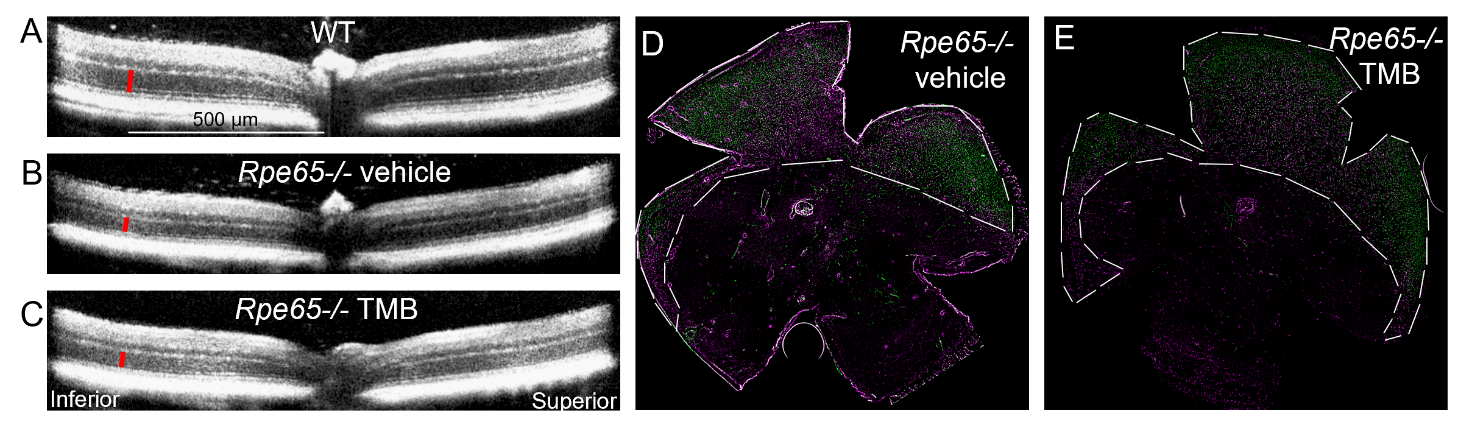
**

Supplementary Figure 8. Representative OCT and retinal flat-mount images from the study of the Rpe65-/- mouse. Figure relates to main Figure 7. (**A**-**C**) Representative OCT images of each group. The red vertical line at 500 µm from ONH illustrates the measure of the outer-retina thickness. (**D**-**E**) Representative retina whole mounts stained with anti S- (violet) and anti M-opsin (green) antibodies; (**D**) from vehicle-treated mice, and (**E**) from TMB-treated *Rpe65^-/-^* mice. Dashed white lines mark the areas of remaining cone density. This image was cropped using ImageJ and used for the semi-automated M-cone count presented in Figure 7L.

**
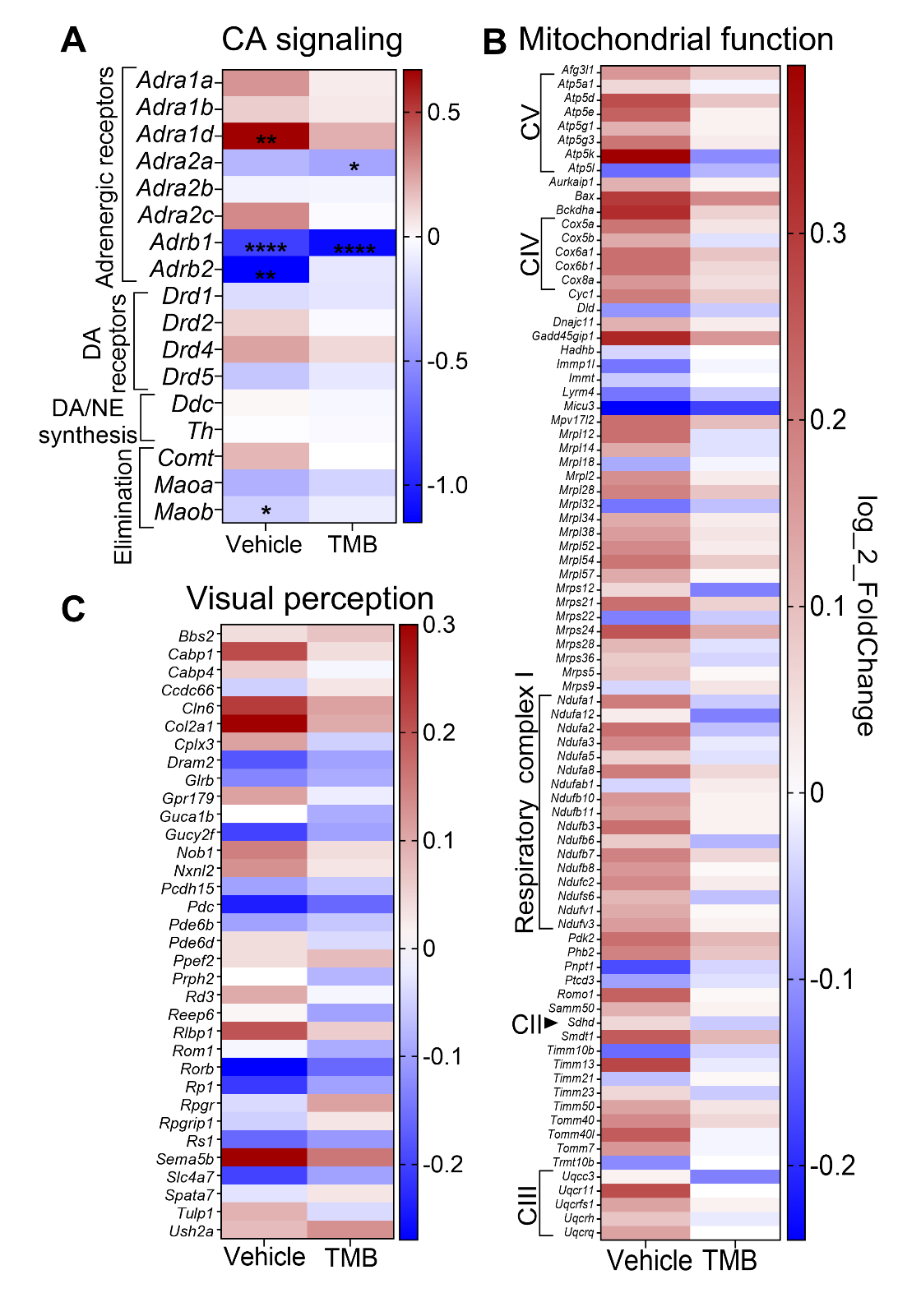
**

Supplementary Figure 9. Bulk RNA-seq in Rpe65-/- and WT retinas indicates transcriptomic stabilization closer to healthy levels with TMB treatment. Data relates to main Figure 7M. (**A**) Expression heatmap showing regulation difference compared to WT for genes encoding adrenergic and dopamine receptors, as well as major catecholamine synthetizing or degrading enzymes. The asterisks signify significantly regulated genes compared to WT: **P* < 0.05, ***P* < 0.01, *****P* < 0.0001. (**B**) Expression heatmap showing regulation difference compared to WT for genes involved in gene set enrichment analysis (GSEA) term ´Visual perception´. (**C**) Expression heatmap showing regulation difference compared to WT for genes involved in GSEA term ´Mitochondrial protein containing complex´.

**
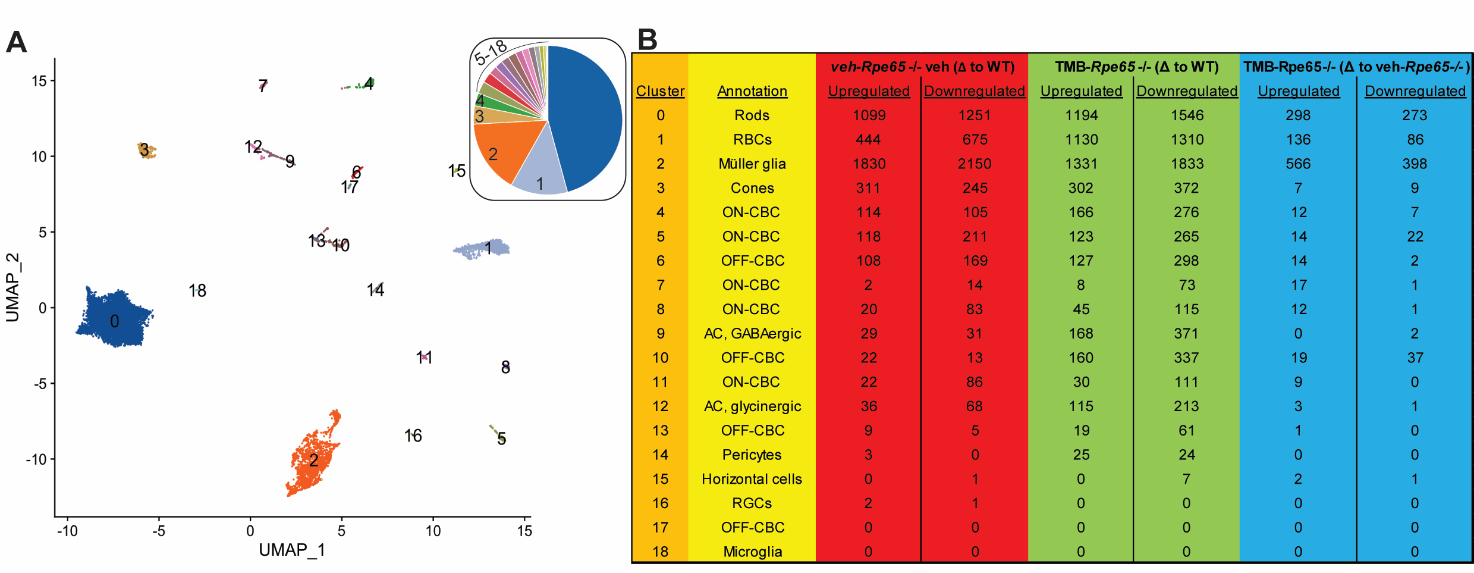
**

Supplementary Figure 10. Single-cell RNA sequencing in Rpe65-/- mouse retinas indicate extensive transcriptomic regulation particularly in the Müller glia cells. Data relates to the main Figure 7P-R. (**A**) Group-consolidated UMAPs by cluster. Treatment groups were combined for generation of the UMAPs. The insert shows cluster size breakout as a pie chart. Single-cell suspensions were prepared from seven *Rpe65^-/-^* mice per group, whereas WT mouse group consisted of three mice. Analysis was performed from 4135 cells from WT mice, 3979 cells from vehicle-treated *Rpe65*^-/-^ mice, and 5901 cells from TMB-treated *Rpe65*^-/-^ mice. (**B**) Table showing the number of differentially expressed (DE) genes for all clusters and for all groupwise comparisons. The cutoff for DE genes was set at P < 0.05 after adjustment for multiple comparisons. RBCs, rod bipolar cells; ON-CBC, ON cone bipolar cells; OFF-CBC, OFF cone bipolar cells; ACs, amacrine cells; RGCs, retinal ganglion cells.


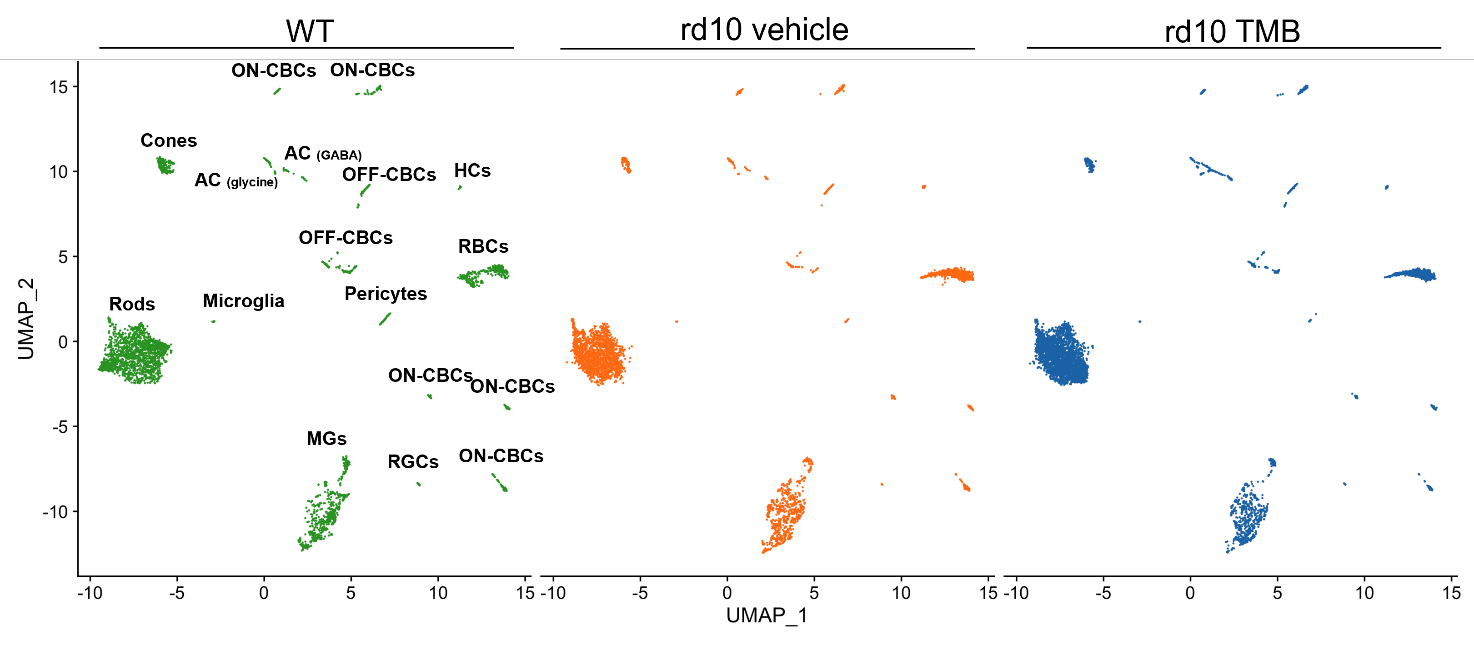


Supplementary Figure 11. Single-cell transcriptome UMAPs separately for each group in the Rpe65-/- mouse dataset. Note the different appearance of the Müller cell cluster between groups. Data relates to the main Figure 7P-R. RBCs, rod bipolar cells; ON-CBC, ON cone bipolar cells; OFF-CBC, OFF cone bipolar cells; ACs, amacrine cells; HCs, horizontal cells; MGs, Müller glia cells; RGCs, retinal ganglion cells.


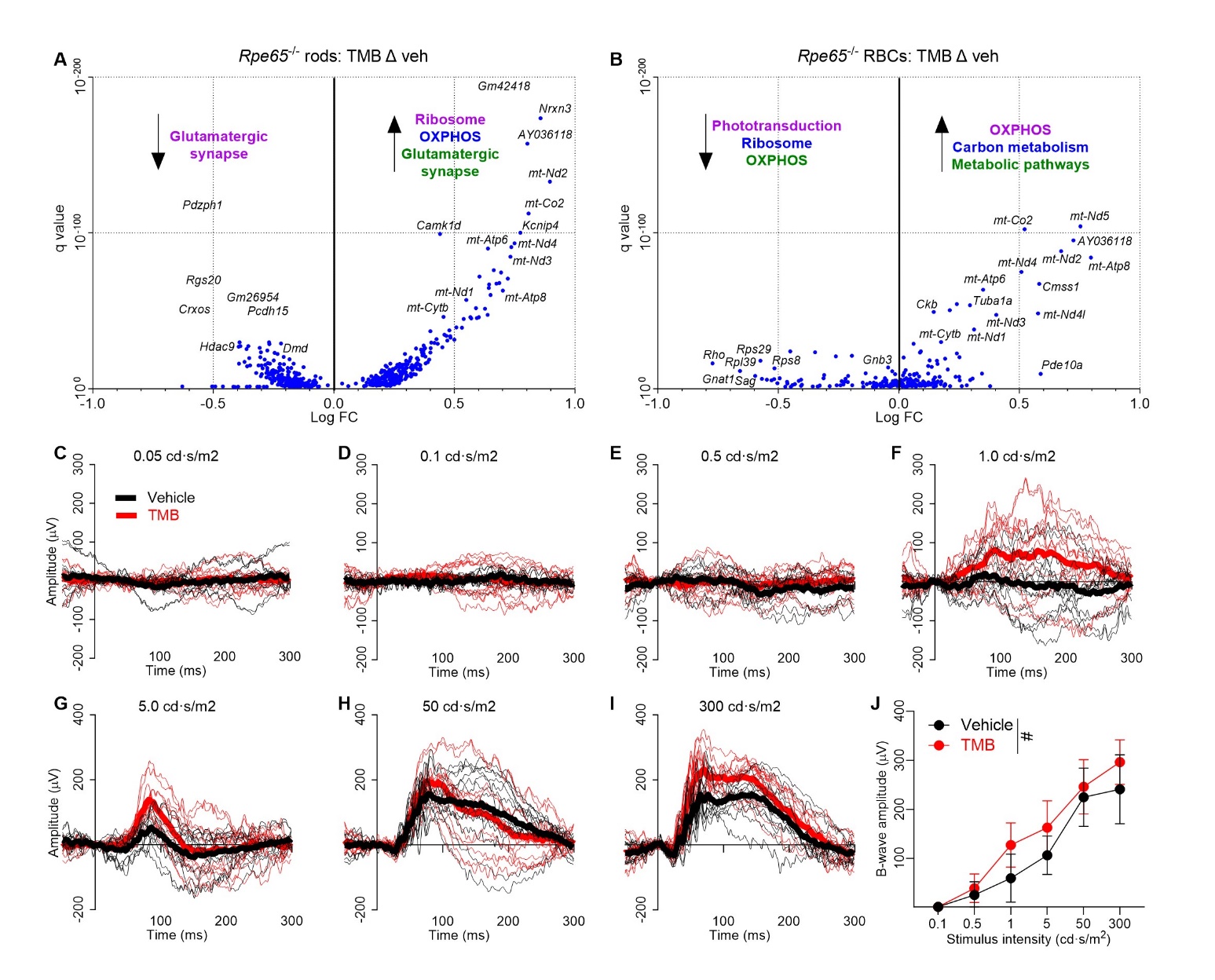


Supplementary Figure 12. TMB treatment enhances mitochondrial gene expression in Rpe65-/- mouse rods and RBCs, and improves rod-mediated ERG responses. Data relates to the main Figure 7. *Rpe65^-/-^* mice were fed with vehicle- or TMB diets for 5 weeks starting at P21. Doses in the TMB pellets were: tamsulosin 50 parts per million (ppm) / 50 mg compound per 1 kg of pellet, metoprolol 2500 ppm / 2500 mg compound per 1 kg of pellet, bromocriptine 250 pm / 250 mg compound per 1 kg of pellet. (**A**-**B**) Volcano plots of DE genes in rods (**A**) and rod bipolar cells (**B**, RBCs) from scRNA-seq data. Relevant down- or upregulated KEGG pathways are highlighted. (**C**-**I**). Scotopic ERG waveforms. Thin lines present individual responses and thick lines group-averages. (**J**) B-wave amplitude analysis. This ERG recording was performed on the same mice that we used for generation of the scRNA-seq data. Recording was performed at P52-P55, mice were allowed to recover from anesthesia for five days, and samples for scRNA-seq were collected at P57-P60. The statistical analysis was performed with repeated measures ANOVA with Greenhouse-Geisser correction, and the pound sign indicates significant between-subjects effect: ^#^*P* < 0.05.


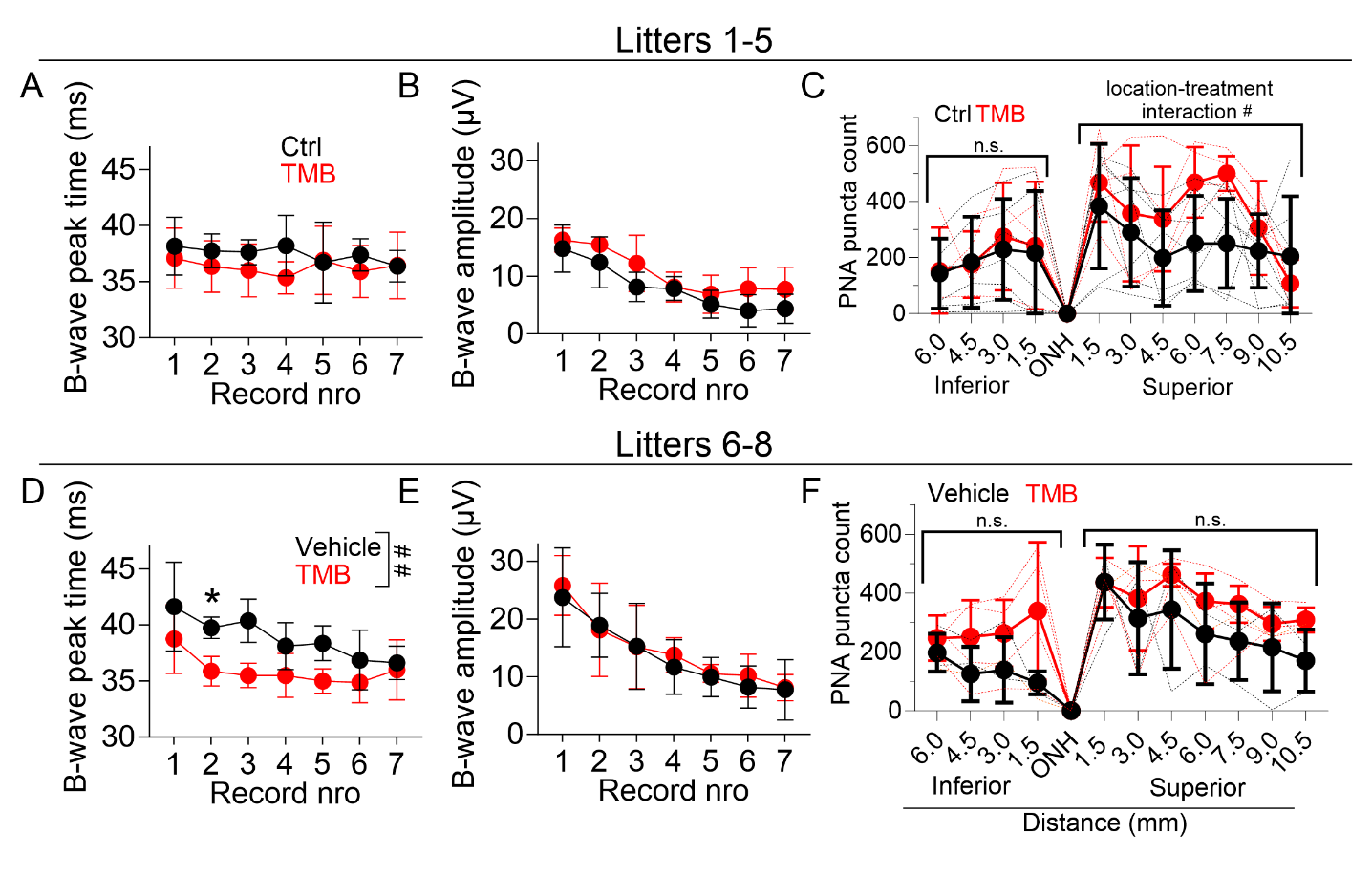


Supplementary Figure 13. Drug efficacy-evaluation parameters do not differ substantially between dog litters (1-5 vs. 6-8). Data relates to the main Figure 9. Note that different study designs were used for the two sets of litters (1-5 and 6-8). For litters 1-5, the control dogs were untreated, and the TMB-treated dogs received one infusion pump per surgery. For litters 6-8, the control dogs were vehicle-treated, and the TMB-treated dogs received one infusion pump per surgery for the first two months of the study, and thereafter dosing was increased to two pumps per surgery. See details in Material & Methods. (**A-C**) ERG b-wave peak time (**A**), b-wave amplitude (**B**), and PNA puncta counts (**C**) in litters 1-5. (**D-F**) ERG b-wave peak time (**D**), b-wave amplitude (**E**), and PNA puncta counts (**F**) in litters 6-8. PNA count analysis was performed separately for the inferior and superior retina sides. Statistical analysis was performed by repeated measures (ERGs and superior retina PNA counts) or mixed effects model (inferior retina PNA counts due to two missing parameters) two-way ANOVA followed by Bonferroni post hoc tests. The pound sign signifies a significant between subjects-within subjects interaction; ^#^*P* < 0.05 (in C), or between-subjects main effect; ^##^*P* < 0.01 (in D). The asterisk * signifies *P* < 0.05 in the Bonferroni test. Data are presented as mean ± SD. Consolidated data from litters 1-5 and 6-8 was used for analyses in main Figure 9E, F, J, and L.


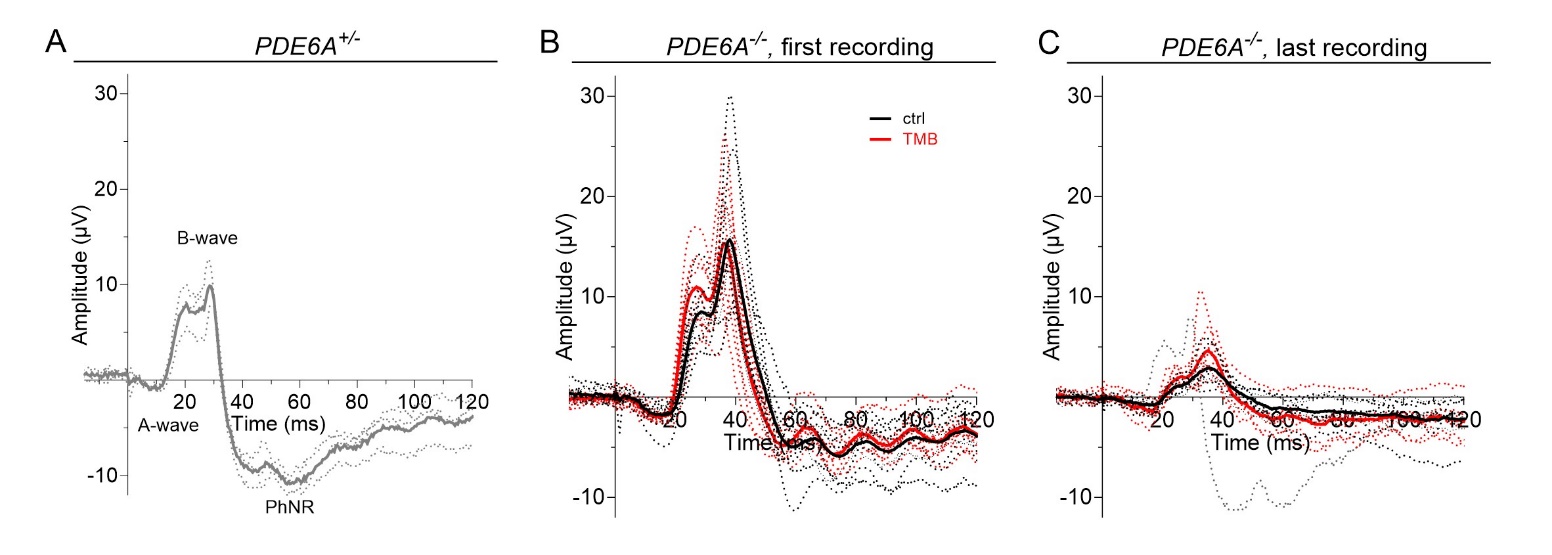


Supplementary Figure 14. ERG waveforms in heterozygote PDE6A carrier dogs (A), and in homozygote PDE6A dogs during the first (B) and last (C) recording session. Data is reproduced from main Figure 9D. (**A**) The *PDE6A^+/-^* dogs (grey, n=3) were recorded only once, when each dog was ~6, 12, or 12.5 months old. Thick lines represent group averages, and dashed thin lines correspond to individual dog responses. (**B**) At the time of first recording, the *PDE6A^-/-^* dogs were 7-8 weeks old (black = controls (n=10); red = TMB-treated (n=9)). (**C**) ERG recordings during the last recording (6 months after the first) show greatly diminished cone-ERG amplitudes for the *PDE6A^-/-^* dogs. During the last recordings, *PDE6A^-/-^* dogs were ~7.5 months old.


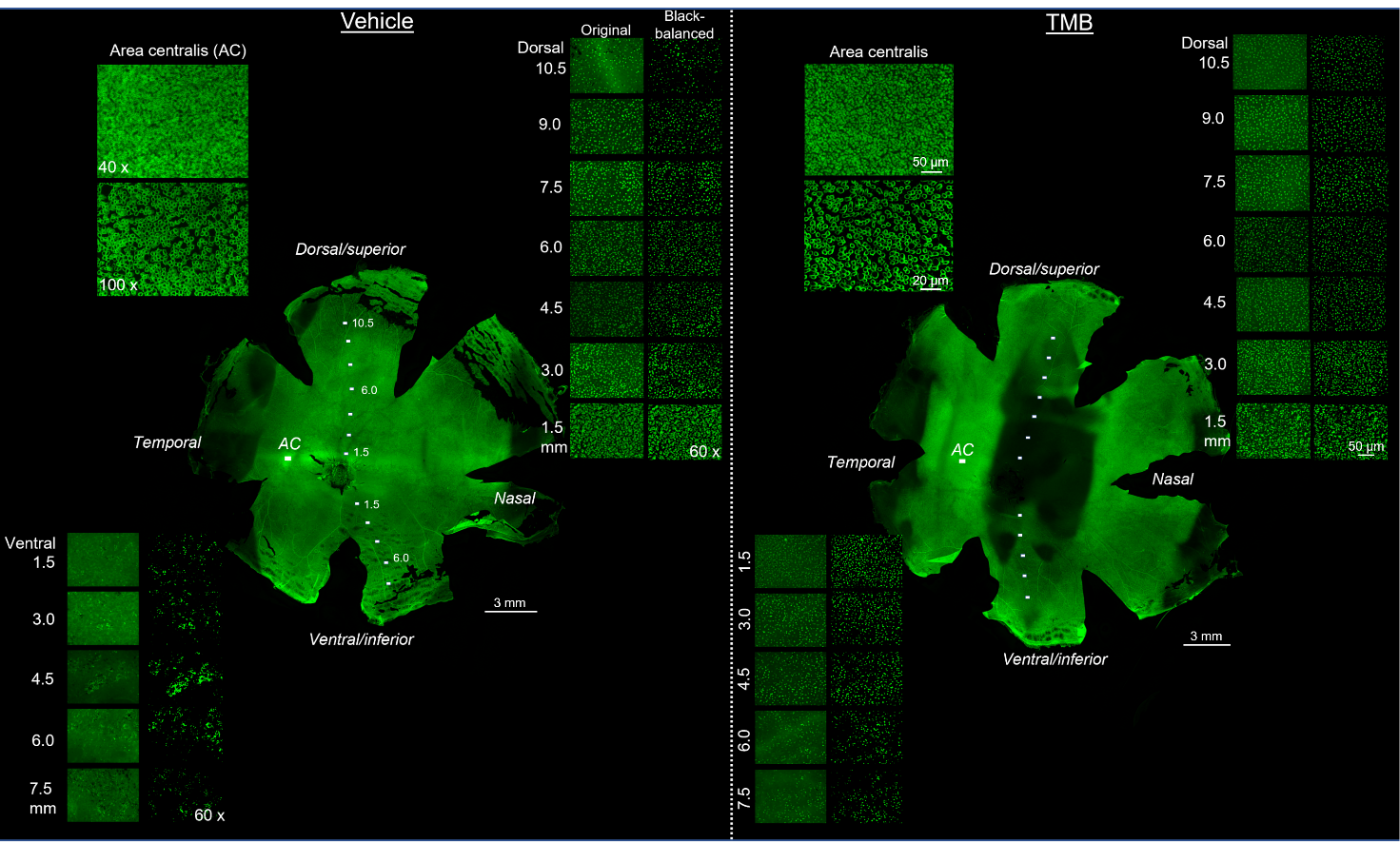


Supplementary Figure 15. PNA puncta-counting method for flat mounts of dog retinas. Data is reproduced from main Figure 9G-I, K. Representative example of a whole mount of a dog retina stained with peanut agglutinin (PNA), a cone marker. PNA punctate-count data for Figure 8J was acquired from images obtained with a 60x objective, using maximum intensity projections of z-stacks. The original images were black-balanced to remove excess background signal before counting. Imaging and counting were performed manually by an experimenter blinded to the treatments. PNA punctate-count data at AC (main Figure 8L) was acquired from images obtained with a 100x objective, using the optical sectioning tool of a Keyence BZ-X800 microscope. Note that the “shadows” in the whole mounts are not a sign of degeneration; rather they are technical glitches caused by folding of the large dog retinas during processing for immunohistochemistry. As evidenced from the TMB sample, these areas can be sampled equally well.


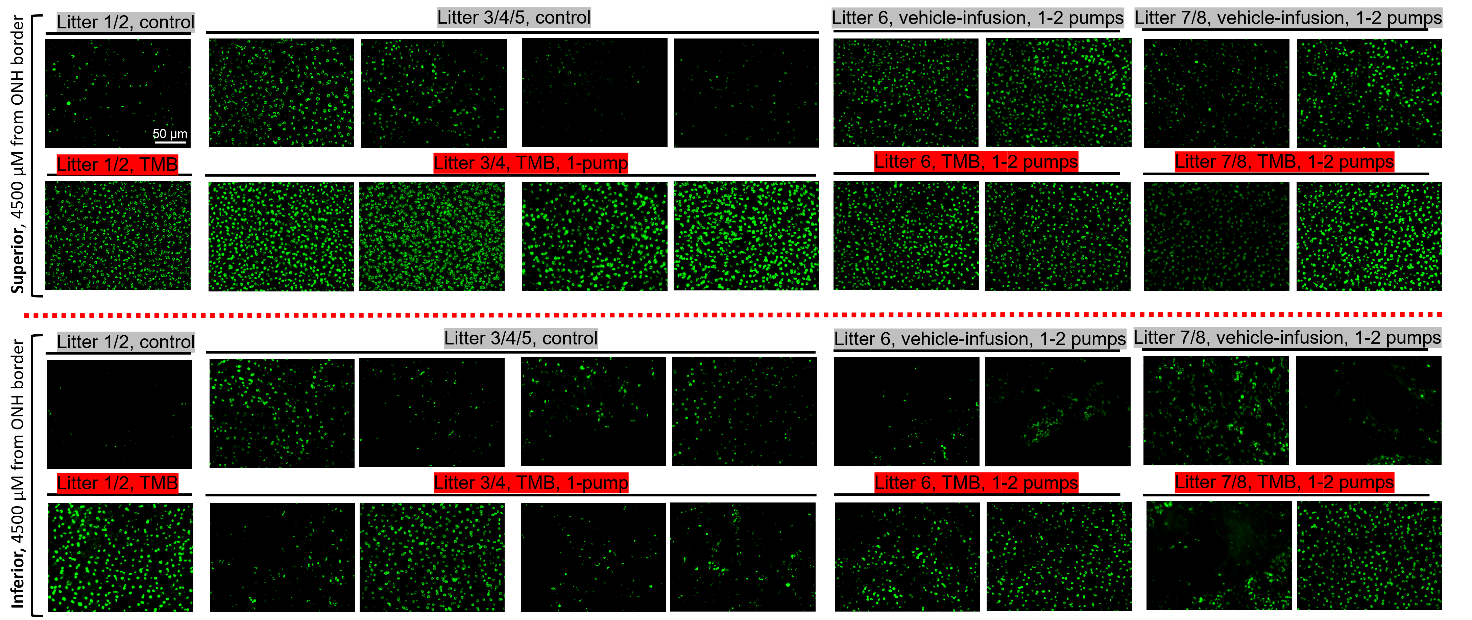


Supplementary Figure 16. Dorsal and ventral middle-retina images, for dog flat mounts centered at 4500 µm from the optic nerve head. Data is partially reproduced from main Figure 9H-I. The dorso-ventral orientation was confirmed from whole mount images before imaging of the PNA punctate-counting windows. Z-stack images were captured using a 60x objective. Maximum intensity projections were processed for counting, and to obtain representative images.


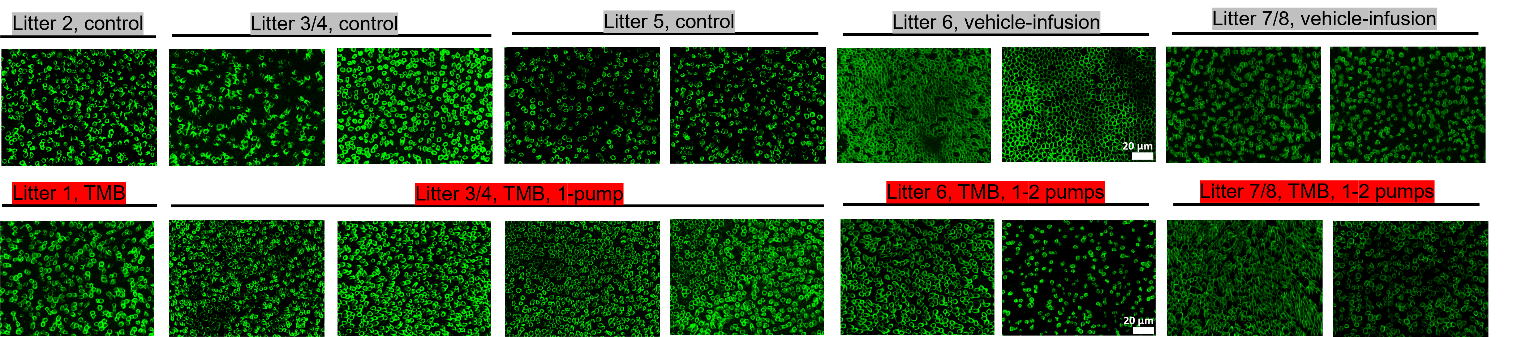


Supplementary Figure 17. Area centralis images from dog flat mounts that were used for PNA punctate counting. Data is partially reproduced from main Figure 9K. The area centralis (AC) is densely populated by cone photoreceptors, mimicking the human macula. The AC appears temporally and slightly dorsally ~1.5-3 mm away from the optic nerve head (ONH) in dogs. The location was oriented using whole mount images (see Figure S15), and highest cone density was confirmed online before capturing the images. Imaging was centered at 2.5 mm from the ONH (or the closest vicinity, if there was any tissue disruption). Z-stacks were acquired using a 100x objective, and 0.2 µm intervals between images. The optical sectioning tool of a Keyence BZ-X800 microscope was utilized to obtain high-resolution.

# Supplementary Table 1. List of antibodies used in the study.

| **Primary antibody** | **Dilution, IHC** | **Dilution, WB** | **Source** | **Cat. No.** |
| --- | --- | --- | --- | --- |
| Rabbit anti-M opsin | 1:1000 | - | Novus Biologicals | NB110-74730 |
| Goat anti-S opsin | 1:2000 | - | Bethyl Laboratories (custom) | n.a. |
| Rabbit anti-alpha tubulin | - | 1:2000 | Cell Signaling Technology | 2144S |
| Rabbit anti-cone arrestin | - | 1:1000 | Millipore-Merck | AB15282 |
| Rabbit anti-SOD2 | - | 1:5000 | Abcam | ab13533 |
| Rabbit anti-GAPDH | - | 1:20 000 | Proteintech | 10494-1-AP |
| Mouse anti-rhodopsin (1D4) | - | 1:2000 | Made in-house | n.a. |
| Mouse anti-catalase | - | 1:500 | Santa Cruz Biotechnology | sc-271803 |
| Mouse anti-GFAP | - | 1:5000 | Cell Signaling Technology | 3670 |
| Mouse anti-COMT | - | 1:2000 | BD Transduction Laboratories | 611970 |
| Biotinylated Peanut Agglutinin | 1:1000 | - | Sigma-Aldrich | L6135 |
| **Secondary antibody** |  |  |  |  |
| Donkey anti-rabbit AlexaFluor 647 | 1:1000 | - | Abcam | ab150075 |
| Donkey anti-goat AlexaFluor 488 | 1:1000 | - | Abcam | ab150129 |
| Goat anti-rabbit IRDye 800CW | - | 1:10 000 | LI-COR Biosciences | 926-32211 |
| Goat anti-mouse IRDye 680RD | - | 1:10 000 | LI-COR Biosciences | 926-68070 |
| Streptavidin, Alexa Fluor 488 conjugate | 1:1000 | - | Invitrogen | S11223 |

# Supplementary Table 2. The detection of drugs with mass spectrometry.

|  | Precursor ion (m/z) | Transition ion (m/z) | Normalized Collision Energy |
| --- | --- | --- | --- |
| **Metoprolol** | 268.2 | 191.1 | 29 |
| ***d*_7_-Metoprolol** | 275.1 | 191 | 29 |
| **Tamsulosin** | 409.2 | 270.8 | 23 |
| ***d*_5_-Tamsulosin** | 414.2 | 228.0 | 23 |
| **Bromocriptine** | 654.3 | 346.1 | 26 |
| **α-Ergocryptine** | 576.3 | 268.0 | 26 |
