# Supplementary material for: A combination treatment based on drug repurposing demonstrates mutation-agnostic efficacy in pre-clinical retinopathy models
Source: Nat Commun. 2024 Jul 15;15:5943. doi: 10.1038/s41467-024-50033-5 (PMC11251169; doi:10.1038/s41467-024-50033-5)
Supplement: Supplementary file 3 — Description Of Additional Supplementary File [file 41467_2024_50033_MOESM3_ESM.pdf]

Description of Additional supplementary files

Supplementary Data 1: Parameters of study groups in each experiment

Supplementary Data 2: LC-MS/MS peak data and calibrations for drug level measurements

Supplementary Data 3: Rd10 mouse retina bulk RNA-seq VENN diagram inputs

Supplementary Data 4: Rd10 mouse retina bulk RNA-seq: read count table of top 5000 most expressed nuclear genes.

Supplementary Data 5: Rd10 mouse retina bulk RNA-seq: read count table of mitochondrially encoded genes.

Supplementary Data 6: Rd10 mouse retina bulk RNA-seq: expression of retinitis pigmentosa-associated genes

Supplementary Data 7: Rd10 mouse retina scRNA-seq, all clusters: WT versus rd10-vehicle groups

Supplementary Data 8: Rd10 mouse retina scRNA-seq, all clusters: WT versus rd10-TMB groups

Supplementary Data 9: Rd10 mouse retina scRNA-seq, all clusters: rd10-vehicle versus rd10-TMB groups

Supplementary Data 10: Rpe65-KO mouse retina bulk RNA-seq: read count table of top 5000 most expressed nuclear genes.

Supplementary Data 11: Rpe65-KO mouse retina bulk RNA-seq VENN diagram inputs

Supplementary Data 12: Rpe65-KO mouse retina bulk RNA-seq: read count table of mitochondrially encoded genes.

Supplementary Data 13: Rpe65-KO mouse retina scRNA-seq, all clusters: WT versus Rpe65-KO-vehicle groups

Supplementary Data 14: Rpe65-KO mouse retina scRNA-seq, all clusters: WT versus Rpe65-KO-TMB groups

Supplementary Data 15: Rpe65-KO mouse retina scRNA-seq, all clusters: Rpe65-KO-vehicle versus Rpe65-KO-TMB groups
