# Supplementary material for: A combination treatment based on drug repurposing demonstrates mutation-agnostic efficacy in pre-clinical retinopathy models
Source: Nat Commun. 2024 Jul 15;15:5943. doi: 10.1038/s41467-024-50033-5 (PMC11251169; doi:10.1038/s41467-024-50033-5)
Supplement: Supplementary file 19 — Reporting summary [file 41467_2024_50033_MOESM19_ESM.pdf]

nature portfolio

Corresponding author(s): Henri Leinonen

Last updated by author(s): Jun 7, 2024

Reporting Summary

Nature Portfolio wishes to improve the reproducibility of the work that we publish. This form provides structure for consistency and transparency in reporting. For further information on Nature Portfolio policies, see our Editorial Policies and the Editorial Policy Checklist.

Statistics

For all statistical analyses, confirm that the following items are present in the figure legend, table legend, main text, or Methods section.

n/a	Confirmed	
☐	☒	The exact sample size (*n*) for each experimental group/condition, given as a discrete number and unit of measurement
☒	☐	A statement on whether measurements were taken from distinct samples or whether the same sample was measured repeatedly
☐	☒	The statistical test(s) used AND whether they are one- or two-sided<br>*Only common tests should be described solely by name; describe more complex techniques in the Methods section.*
☒	☐	A description of all covariates tested
☐	☒	A description of any assumptions or corrections, such as tests of normality and adjustment for multiple comparisons
☐	☒	A full description of the statistical parameters including central tendency (e.g. means) or other basic estimates (e.g. regression coefficient) AND variation (e.g. standard deviation) or associated estimates of uncertainty (e.g. confidence intervals)
☐	☒	For null hypothesis testing, the test statistic (e.g. *F*, *t*, *r*) with confidence intervals, effect sizes, degrees of freedom and *P* value noted<br>*Give P values as exact values whenever suitable.*
☒	☐	For Bayesian analysis, information on the choice of priors and Markov chain Monte Carlo settings
☒	☐	For hierarchical and complex designs, identification of the appropriate level for tests and full reporting of outcomes
☒	☐	Estimates of effect sizes (e.g. Cohen's *d*, Pearson's *r*), indicating how they were calculated

Our web collection on statistics for biologists contains articles on many of the points above.

Software and code

Policy information about availability of computer code

Data collection	Xcalibur, Diagnosys Espion V6, Clampex 10.6, Bioptigen spectral-domain OCT, Psychophycis Toolbox (version 3) for MatLab 2021, Scout recording system, Odyssey XF Imaging System, Phenosys qOMR, Keyence BZ-X800, Illumina HiSeq, Nanodrop and Agilent 2100 devices (QC), 10× Genomics Chromium, Illumina NovaSeq6000
Data analysis	Image J 1.47v, Keyence BZ-X800 Analyzer software, Diagnosys Espion V6, MatLab 2021, Clampfit 10.6, FastQC v0.11.9, Trimmomatic v0.40, STAR aligner 2.7.11b, RseQC v5.0.1, HTSeq 2.0, R v4.2.1., clusterProfiler v4.7.2, bclfastq v2.20, Cell Ranger v6.0.1, Cumulus software v1.5.0, Scrublet v0.2.1, Terra Cloud Platform, Seurat 3.2.2, Harmony package v0.1.0, GraphPad Prism version 10, Adope Illustrator and Photoshop 2022, Microsoft Excel for Microsoft 365 MSO (Version 2405)

For manuscripts utilizing custom algorithms or software that are central to the research but not yet described in published literature, software must be made available to editors and reviewers. We strongly encourage code deposition in a community repository (e.g. GitHub). See the Nature Portfolio guidelines for submitting code & software for further information.

Data

Policy information about availability of data

All manuscripts must include a data availability statement. This statement should provide the following information, where applicable:

- Accession codes, unique identifiers, or web links for publicly available datasets
- A description of any restrictions on data availability
- For clinical datasets or third party data, please ensure that the statement adheres to our policy

The bulk RNA-seq and scRNA-seq data generated in this study have been deposited in the NCBI GEO database under accession code GSE238218. The raw and processed data generated in this study are provided in the Supplementary Information/Supplementary Data files/Source Data file.

Research involving human participants, their data, or biological material

Policy information about studies with human participants or human data. See also policy information about sex, gender (identity/presentation), and sexual orientation and race, ethnicity and racism.

Reporting on sex and gender	*Use the terms sex (biological attribute) and gender (shaped by social and cultural circumstances) carefully in order to avoid confusing both terms. Indicate if findings apply to only one sex or gender; describe whether sex and gender were considered in study design; whether sex and/or gender was determined based on self-reporting or assigned and methods used. Provide in the source data disaggregated sex and gender data, where this information has been collected, and if consent has been obtained for sharing of individual-level data; provide overall numbers in this Reporting Summary. Please state if this information has not been collected. Report sex- and gender-based analyses where performed, justify reasons for lack of sex- and gender-based analysis.*
Reporting on race, ethnicity, or other socially relevant groupings	*Please specify the socially constructed or socially relevant categorization variable(s) used in your manuscript and explain why they were used. Please note that such variables should not be used as proxies for other socially constructed/relevant variables (for example, race or ethnicity should not be used as a proxy for socioeconomic status). Provide clear definitions of the relevant terms used, how they were provided (by the participants/respondents, the researchers, or third parties), and the method(s) used to classify people into the different categories (e.g. self-report, census or administrative data, social media data, etc.) Please provide details about how you controlled for confounding variables in your analyses.*
Population characteristics	*Describe the covariate-relevant population characteristics of the human research participants (e.g. age, genotypic information, past and current diagnosis and treatment categories). If you filled out the behavioural & social sciences study design questions and have nothing to add here, write "See above."*
Recruitment	*Describe how participants were recruited. Outline any potential self-selection bias or other biases that may be present and how these are likely to impact results.*
Ethics oversight	*Identify the organization(s) that approved the study protocol.*

Note that full information on the approval of the study protocol must also be provided in the manuscript.

Field-specific reporting

Please select the one below that is the best fit for your research. If you are not sure, read the appropriate sections before making your selection.

☒ Life sciences ☐ Behavioural & social sciences ☐ Ecological, evolutionary & environmental sciences

For a reference copy of the document with all sections, see nature.com/documents/nr-reporting-summary-flat.pdf

Life sciences study design

All studies must disclose on these points even when the disclosure is negative.

Sample size	No sample-size calculation was used to predetermine sample sizes. The study was exploratory in nature and a sufficient enough amount of biological replicates (animals) were used to assess statistical significance, in keeping with 3Rs of animal research (reduction). The sample sizes have been chosen in accordance with the standards in the field.
Data exclusions	The only data points excluded were in Figure 8B showing LC-MS-quantification of drug serum level in dogs. These outliers were detected using GraphPad Prism´s ROUT method with false discovery rate set at 0.5 %. Some electrophysiology (ERG) recordings could not be accepted for analysis due to noisy signal. All samples that were allocated for immunohistochemisry-based analyses could not be used due to poor sample or staining quality.
Replication	All experiments involving animals were performed in at least three independent animals, with reproducible results. Immunoblotting experiments were not replicated.
Randomization	The animals were randomly assigned to control and experimental groups. For all studies, samples and organisms were randomly allocated to

Randomization	experimental groups.
Blinding	Full blinding was possible andused in the following data collection: all dog studies, LC-MS/M, microscopy.<br>For mouse in vivo studies, blinding was impractical because of dietary drug administration and drug pellet being color-coded. In addition, the same experimenter performed all the in vivo studies. Blinding was however possible and used in following data analyses: RNA-seq, scRNA-seq, retina/eye morphometry (ONL thickness, RPE detachment), and cell counting.

Reporting for specific materials, systems and methods

We require information from authors about some types of materials, experimental systems and methods used in many studies. Here, indicate whether each material, system or method listed is relevant to your study. If you are not sure if a list item applies to your research, read the appropriate section before selecting a response.

Materials & experimental systems

n/a	Involved in the study	
☐	☒	Antibodies
☒	☐	Eukaryotic cell lines
☒	☐	Palaeontology and archaeology
☐	☒	Animals and other organisms
☒	☐	Clinical data
☒	☐	Dual use research of concern
☒	☐	Plants

Methods

n/a	Involved in the study	
☒	☐	ChIP-seq
☒	☐	Flow cytometry
☒	☐	MRI-based neuroimaging

Antibodies

Antibodies used	Rabbit anti-M opsin (Novus Biologicals NB110-74730), Goat anti-S opsin (Bethyl laboratories, custom), Rabbit anti-alpha tubulin (CST, 2144S), Rabbit anti-cone arrestin (Abcam, ab15282), Rabbit anti-SOD2 (Abcam, ab13533), Rabbit anti-GAPDH (Proteintech 10494-1-AP), Mouse anti-rhodopsin (1D4, custom), Mouse anti-catalase (SantaCruz Biotech, sc-271803), Mouse anti-GFAP (CST, 3670), Mouse anti-COMT (BD transduction laboratories, 611970), Biotinylated PNA (Sigma-Aldrich, L6135), Donkey anti-rabbit AlexaFluore 647 (Abcam, ab150075), Donkey anti-goat AlexaFluore 488 (Abcam, ab150129), Goat anti-rabbit IRDye 800CW (Li-Cor, 926-32211), Goat anti-mouse IRDye 680RD (Li-Cor, 926-68070), Streptavidin Alexa Fluor 488 conjugate (InVitrogen, S11223)
Validation	Each antibody was validated for species and application, as appropriate, on the manufacturer's website, as supported by relevant citations on the product pages. S-opsin and M-opsin antibodies were validated by a previous paper (Leinonen et al., Invest Ophthalmol Vis Sci, 2019). For cone arrestin, the website states "Anti-Cone Arrestin, Cat. No. AB15282, is a highly specific rabbit polyclonal antibody that targets Arrestin-C and has been tested in Immunohistochemistry (Paraffin) and Western Blotting. The 1D4 antibody used is a field-standard for the use of rhodopsin studies.

Animals and other research organisms

Policy information about studies involving animals; ARRIVE guidelines recommended for reporting animal research, and Sex and Gender in Research

Laboratory animals	C57BL/6J mice (RRID:IMSR_JAX:000664), one to three months old.<br>Pde6betaRd10 (rd10) mice (RRID:IMSR_JAX:004297), one to four months old. C5757/6J background.<br>RhoP23H/WT (P23H) mice (RRID: IMSR_JAX:017628). From three weeks to eights months old. C5757/6J background.<br>Rpe65-/- mice (a kind gift from Dr. Michael Redmond, National Institutes of Health, Bethesda, MD). From three weeks to two months old. These mouse strain exact background is not known, but it is likely C57BL/6J dominant and it´s coloration is black.<br>PDE6A-/- dog. The PDE6A mutation was a spontaneous mutation identified in the Cardigan Welsh Corgi breed. Affected dogs were bred with laboratory beagles to create the colony maintained at Michigan State University. One to eight months old.
Wild animals	The study did not involve the use of wild animals.
Reporting on sex	The data in this report is generally based on data from mice and dogs of both sexes. There was a few exceptions where both sexes were not used. Behavioral optomotor response experiments were performed in male Rpe65-KO mice only due to poor reproducibility of responses in young adult female Rpe65-KO mice in initial experiments. Multi-month experiments in dark-reared rd10 mice and in P23H mice were performed in female mice, as well as scRNA-seq in dark-reared rd10 mice. Instead, scRNA-seq in Rpe65-KO mice was performed in males. The details are provided in the Source Data File and in Supplementary Data 1.
Field-collected samples	The study did not involve samples collected from the field.
Ethics oversight	For all experimental procedures, animal subjects were treated in accordance with the NIH guidelines for the care and use of laboratory animals, the ARVO Statement for the Use of Animals in Ophthalmic and Vision Research, and the European Directive. Most mouse experiments were conducted at the University of California Irvine (UCI). V1 electrophysiology experiments were conducted in Polish Academy of Science, Warsaw, Poland, and bulk RNA-seq analyses at John Hopkins University. All protocols used in experiments on mice have been approved by the Institutional Animal Care and Use Committee (IACUC) at the UCI (protocol #AUP-21-096), or a

permission was approved by the 1st Local Ethical Committee in Warsaw under the number 1400P2/2022. The dog experiments were conducted at Michigan State University (MSU) and were approved by the IACUC at MSU (# 05/14-090-00, # 05/17-075-00, and # PROTO202000013).

Note that full information on the approval of the study protocol must also be provided in the manuscript.

Plants

Seed stocks

Report on the source of all seed stocks or other plant material used. If applicable, state the seed stock centre and catalogue number. If plant specimens were collected from the field, describe the collection location, date and sampling procedures.

Novel plant genotypes

Describe the methods by which all novel plant genotypes were produced. This includes those generated by transgenic approaches, gene editing, chemical/radiation-based mutagenesis and hybridization. For transgenic lines, describe the transformation method, the number of independent lines analyzed and the generation upon which experiments were performed. For gene-edited lines, describe the editor used, the endogenous sequence targeted for editing, the targeting guide RNA sequence (if applicable) and how the editor was applied.

Authentication

Describe any authentication procedures for each seed stock used or novel genotype generated. Describe any experiments used to assess the effect of a mutation and, where applicable, how potential secondary effects (e.g. second site T-DNA insertions, mosiacism, off-target gene editing) were examined.
